# Comparison between audio-vestibular findings and contrast-enhanced MRI of inner ear in patients with unilateral Ménière’s disease

**DOI:** 10.3389/fnins.2023.1128942

**Published:** 2023-03-13

**Authors:** Yangming Leng, Wenliang Fan, Yingzhao Liu, Kaijun Xia, Renhong Zhou, Jingjing Liu, Hongchang Wang, Hui Ma, Bo Liu

**Affiliations:** ^1^Department of Otorhinolaryngology Head and Neck Surgery, Union Hospital, Tongji Medical College, Huazhong University of Science and Technology, Wuhan, China; ^2^Department of Radiology, Union Hospital, Tongji Medical College, Huazhong University of Science and Technology, Wuhan, China; ^3^Hubei Province Key Laboratory of Molecular Imaging, Wuhan, China

**Keywords:** Ménière’s disease, endolymphatic hydrops, magnetic resonance imaging, glycerol test, caloric test, vestibular evoked myogenic potentials, electrocochleogram, video head impulse test (vHIT)

## Abstract

**Objective:**

The diagnosis of Ménière’s disease (MD), characterized by idiopathic endolymphatic hydrops (ELH), remains a clinical priority. Many ancillary methods, including the auditory and vestibular assessments, have been developed to identify ELH. The newly emerging delayed magnetic resonance imaging (MRI) of the inner ear after intratympanic gadolinium (Gd) has been used for identifying ELH *in vivo*. We aimed to investigate the concordance of audio-vestibular and radiological findings in patients with unilateral MD.

**Methods:**

In this retrospective study, 70 patients with unilateral definite MD underwent three-dimensional fluid-attenuated inversion recovery (3D-FLAIR) sequences following intratympanic application of Gd. Audio-vestibular evaluations were performed, including pure tone audiometry, electrocochleogram (ECochG), glycerol test, caloric test, cervical and ocular vestibular evoked myogenic potentials (VEMPs), and video head impulse test (vHIT). The relationship between imaging signs of ELH and audio-vestibular results was investigated.

**Results:**

The incidence of radiological ELH was higher than that of neurotological results, including the glycerol test, caloric test, VEMPs, and vHIT. Poor or slight agreement was observed between audio-vestibular findings and radiological ELH in cochlear and/or vestibular (kappa values <0.4). However, the pure tone average (PTA) in the affected side significantly correlated with the extent of both cochlear (*r* = 0.26795, *p* = 0.0249) and vestibular (*r* = 0.2728, *p* = 0.0223) hydrops. Furthermore, the degree of vestibular hydrops was also positively correlated with course duration (*r* = 0.2592, *p* = 0.0303) and glycerol test results (*r* = 0.3944, *p* = 0.0061) in the affected side.

**Conclusion:**

In the diagnosis of MD, contrast-enhanced MRI of the inner ear is advantageous in detecting ELH over the conventional audio-vestibular evaluations, which estimates more than hydropic dilation of endolymphatic space.

## 1. Introduction

Ménière’s disease (MD) is an inner ear disorder of unknown etiology, which typically manifested as repetitive vertigo attacks in association with fluctuating cochlear symptoms, including sensorineural hearing loss, tinnitus and aural fullness (Merchant et al., 2005). The endolymphatic hydrops (ELH), dilatation of endolymphatic space, is the pathological hallmark of MD, even its role in the pathogenesis and development of this condition is poorly understood (Merchant et al., 2005). Over the years, the diagnosis of MD poses a great challenge to clinicians due to its heterogeneous and fluctuating manifestations. Because the diagnosis of MD relies heavily on symptoms and pure tone audiometry, various neurophysiological techniques have been developed to facilitate its diagnosis. These often included the electrocochleogram (ECochG) (Hornibrook, 2017), glycerol test (Lütkenhöner and Basel, 2013), auditory brainstem responses (Don et al., 2005), caloric/head impulse tests (Hannigan et al., 2021), vestibular evoked myogenic potentials (VEMP) (Welgampola and Colebatch, 2005), etc. However, to date, these ancillary audio-vestibular evaluations are not enrolled in the diagnostic criteria for MD due to their variable sensitivity and specificity.

Recent developments of magnetic resonance imaging (MRI) techniques have allowed the clinicians to visualize ELH *in vivo* using 3 Tesla (3T) scanners and gadolinium (Gd) enhancement by either intratympanic or intravenous application (Nakashima et al., 2007). Multiple techniques and algorithms have been developed to assess the severity of ELH *in vivo*, among which three-dimensional fluid-attenuated inversion recovery (3D-FLAIR) and three-dimensional real inversion recovery (3D-real IR) MRI are recommended as the fundamental imaging sequences (Naganawa et al., 2008; Liu et al., 2022). 3D-real IR MRI could discriminate signals from the endolymphatic space, perilymphatic space, and surrounding bone tissues on one IR image. 3D-FLAIR MRI could better differentiate signal difference between the perilymphatic and endolymphatic space when the Gd concentration is insufficient in the perilymph. Direct visualization of ELH *in vivo* demonstrated by Gd-enhanced MRI of inner ear could provide confirmatory evidence for diagnosing MD, with a sensitivity of 85% and specificity of 92% (Bernaerts et al., 2019), and improve differential diagnosis in patients with suspected MD. It has been well established that radiologically diagnosed hydrops is rather common in healthy populations and other vestibular disorders (van der Lubbe et al., 2020). Meanwhile, 10–32% of patients with MD do not show MRI-demonstrable ELH (Pakdaman et al., 2016). Considering this clinical-radiological inconsistency, imaging evidence of ELH is not mandatory according to the 2020 American Academy of Otolaryngology-Head and Neck Surgery (AAO-HNS) guideline (Basura et al., 2020) and the 2015 Barany Society diagnostic criteria (Lopez-Escamez et al., 2015).

Neurotological tests has been considered as functional indicators of ELH, providing indirect but complementary information in diagnosing MD. There have been many reports addressing the relationship between the MRI-demonstratable ELH and a multitude of neurotology findings. The conclusions were inconsistent due to variability in the evaluation protocols used and the patients included. For example, Yamamoto et al. (2010) and Gürkov et al. (2011) found no consistent correlation between the imaging ELH and the summating potential (SP)/action potential (AP) ratio in extratympanic click ECochG. However, Sun et al. (2021) using a different radiological protocol based on Gd-enhanced MRI of inner ear, demonstrated a significant association between the imaging ELH and the SP/AP ratio. In a recent consensus on MRI evaluation of ELH in MD patients, combined use of Gd-enhanced MRI and audio-vestibular function tests is recommended (Liu et al., 2022). In the diagnostic strategies recently proposed by Japan Society for Equilibrium Research, the vestibular function tests, tests for estimating ELH (such as ECochG, glycerol test, etc.), and MRI imaging of ELH were included in the examination for the diagnosis of MD (Iwasaki et al., 2021). Additionally, contrast-enhanced MRI has been encompassed in this diagnostic criterion for certain MD (Iwasaki et al., 2021). Investigation of the relationship between the MRI-demonstrable ELH and neurotological functional tests may deepen our understanding of inner ear diseases. Therefore, it is necessary to continue to explore the relationship between MRI imaging of ELH and audio-vestibular function in patients with MD.

This study was designed to retrospectively analyze the agreements and correlations between the MRI results of inner ear after intratympanic Gd application and audio-vestibular findings in patients with MD diagnosed clinically. The clinical relevance of radiological and neurotologic results was also explored.

## 2. Materials and methods

### 2.1. Study population

This study recruited 70 patients with unilateral definite MD, who fulfilled the 1995 AAO-HNS diagnostic criteria (Committee on Hearing and Equilibrium, 1995). Exclusion criteria included: (1) infections or malformations in the middle or inner ear; (2) retro-cochlear pathology; (3) comorbidity of MD and vestibular migraine; (4) bilateral MD; (5) history of previous ear surgery or intratympanic injections; and (6) head trauma.

Ethical approval was provided by the ethics committee of Union Hospital, Tongji Medical College of Huazhong University of Science and Technology. This study was performed in compliance with the principles set out in the Declaration of Helsinki. Informed consent was obtained from each patient.

### 2.2. Intratympanic Gd injection and MRI evaluations

Intratympanic Gd injection and MRI examination was performed as previously described (Chen et al., 2012). The Gd-DTPA-dimeglumine solution (MultiHance; Braccosine, Shanghai, China) was used as the contrast agent. Diluted gadolinium hydrate (1:8 with saline) was delivered intratympanically *via* a 23-gauge needle in both ears of each patient. The patient was instructed to maintain head rotation 45°contralaterally for half an hour. Routine and delayed (24 h after Gd administration) 3D-FLAIR MRI images were acquired from each participant using a 3T MRI system (Verio, Siemens, Erlangen, Germany), which was equipped with a 32-channel head coil at Union Hospital, Wuhan, Hubei, China. Routine coronal T2-weighted sequence were obtained to determine the location of the internal auditory canal and to detect the intracranial and cerebellopontine angle lesions. T2 weighted anatomical images as a three-dimensional space sequence and the 3D-SPACE inversion recovery FLAIR sequence were also acquired. The detailed scanning protocol was summarized in Supplementary Table 1. According to the published criteria (Nakashima et al., 2009b), the degree of ELH in the vestibule and cochlea was classified as none, mild, and significant (Table 1). The interval between MRI examination and audio-vestibular tests ranged from 1 to 12 days, which was influenced by the patients’ compliance and arrangement of radiological appointments.

**TABLE 1:** Grading of ELH using magnetic resonance imaging.

Grade of hydrops	Vestibule (area ratio)	Cochlea
None	≤33.3%	No displacement of Reissner’s membrane
Mild	>33.3%, ≤50%	Displacement of Reissner’s membrane, and area of cochlear duct ≤ area of the scala vestibuli
Significant	>50%	Area of cochlear duct exceeds the area of the scala vestibuli

ELH, endolymphatic hydrops.

### 2.3. Audio-vestibular evaluations

The detailed procedures of audio-vestibular evaluations have been described in our previous studies (Leng et al., 2017; Liu et al., 2017), which included the pure tone audiometry, ECochG, glycerol test, caloric test, VEMPs and vHIT. The audio-vestibular tests were performed within an interval of 3 days.

#### 2.3.1. Audiometry

Pure-tone audiometry between 125 Hz and 8000 Hz was conducted in a soundproof cabin. The clinical stage of MD was determined based on three-frequency pure tone average (PTA), calculated as simple arithmetic means of 0.5, 1.0, and 2.0 kHz pure tone threshold. According to the AAO-HNS guideline in 1995 (Committee on Hearing and Equilibrium, 1995), PTA of <26 dB HL was classified as Stage I; PTA of 26–40 dB HL as Stage II; PTA of 41–70 dB HL as Stage III; and PTA of >70 dB HL as Stage IV.

#### 2.3.2. ECochG and audiometric glycerol test

SP and AP were recorded during click evoked ECochG using extra-tympanic electrode. A positive ECochG result was defined as SP/AP ratio ≥0.4, suggestive for ELH. In glycerol test, two patterns of pathological results were noted, i.e., glycerol-induced hearing gain (improvement of hearing ability after glycerol intake) and rebound phenomena (deterioration of hearing ability after glycerol intake) (Matsubara et al., 1984). The result of audiometric glycerol test was deemed positive when the hearing threshold was improved by: (1) ≥10 dB at any three or more frequencies or (2) ≥15 dB at one frequency at any time point after glycerol ingestion.

#### 2.3.3. Caloric test

Bithermal caloric response was measured using infrared videonystagmography (Visual Eyes VNG, Micromedical Technologies, Chatham, IL, USA). Cold (24°C) and warm (50°C) air stimulation was delivered into each external auditory canal alternately, and the maximum slow phase velocity (SPV_max_) of the induced nystagmus was measured after each stimulation. The value of canal paresis (CP) was calculated using the Jongkees formula. Interaural asymmetry of the caloric response ≥25% was taken as evidence of unilateral vestibular hypofunction. Bilateral vestibular hypofunction is considered if SPV_max_ of each ear ≤6°/s after caloric stimulation, or the summated SPVmax ≤20°/s for all four stimulation conditions.

#### 2.3.4. VEMPs

The Eclipse system (Interacoustics A/S, Middelfart, Denmark) was utilized to record cervical VEMP (cVEMP) and ocular VEMP (oVEMP) elicited by air-conducted sound (ACS). Tone bursts (500 Hz, 100 dB nHL, 5 ms, rise-plateau-fall time = 2-1-2 ms) were presented monaurally *via* earphones as stimulus and VEMP response was measured using surface electromyography electrodes. cVEMP response was recorded from ipsilateral sternocleidomastoid muscle (SCM) activated by contralateral head rotation and oVEMP was recorded from contralateral inferior oblique muscles activated by staring in the upward direction. At least 100 stimuli were averaged during each trial. The biphasic waveforms p13-n23 and n10-p15 were recorded and analyzed for cVEMP and oVEMP, respectively. Abnormal cVEMP responses were defined as: (1) the amplitude asymmetry ratio (AR) greater than the mean of normal range ± 2 standard deviation (SD) (the AR ≥36% in our clinic); and (2) absent or decreased amplitude of p13-n23 waveforms. Abnormal oVEMP responses were defined as: (1) no reliable oVEMP response after at least 50 stimuli; and (2) amplitude AR greater than the mean of normal range ± 2 SD (the AR ≥40% in our clinic).

#### 2.3.5. vHIT

The ICS Impulse system (GN Otometrics, Denmark) was used for vHIT. Each patient wore a pair of tightly fitted, lightweight goggles, which were equipped with high-speed camera and can measure the eye movement. The patient was instructed to maintain his gaze at a stationary dot 1 m away. A technician standing behind the patient manually delivered approximately 20 to 25 random, unpredictable, and passive horizontal head impulses (amplitude: 5∼15°, peak velocity: 150∼250°/s, duration: 150–200 ms). Re-fixation saccade with a velocity exceeding 50°/s was considered significant. In this study, pathological vHIT refers to presence of re-fixation saccades with a horizontal vHIT gain <0.8.

### 2.4. Statistical analysis

Data processing and statistical analysis was performed using SPSS R26.0.0.2 software. Continuous variables were presented as median and interquartile interval, and categorical variables were presented as frequency and composition ratios. The correlation between the degree of cochlear and vestibular ELH and the clinical features and audio-vestibular results was assessed by Spearman correlation analysis. The kappa agreement analysis was performed to explore the agreement of radiological results with other test results. The pathological rates of radiological test and other test results were compared by McNemar’s test. A two-sided test with test level *a* = 0.05 was used.

## 3. Results

### 3.1. Demographics

A total of 70 patients with unilateral MD receiving inner ear MRI after intratympanic Gd injection were included. Among them, 28 cases (40%) were male, and 42 cases (60%) were female. Age ranged from 17 to 80 years old (mean 50.7) with a median of 52.5 years and a quartile interval of 18 years. The duration of disease ranged from 1 month to 30 years (mean 3.1 years), with a median of 1.75 years and a quartile interval of 2.5 years. Among these cases, 42 (60%) were left-sided and 28 (40%) were right-sided. Four cases (5.71%) were classified as MD stage I, 14 cases (20%) stage II, 41 cases (58.57%) stage III, and 11 cases (15.71%) stage IV.

**FIGURE 1:**
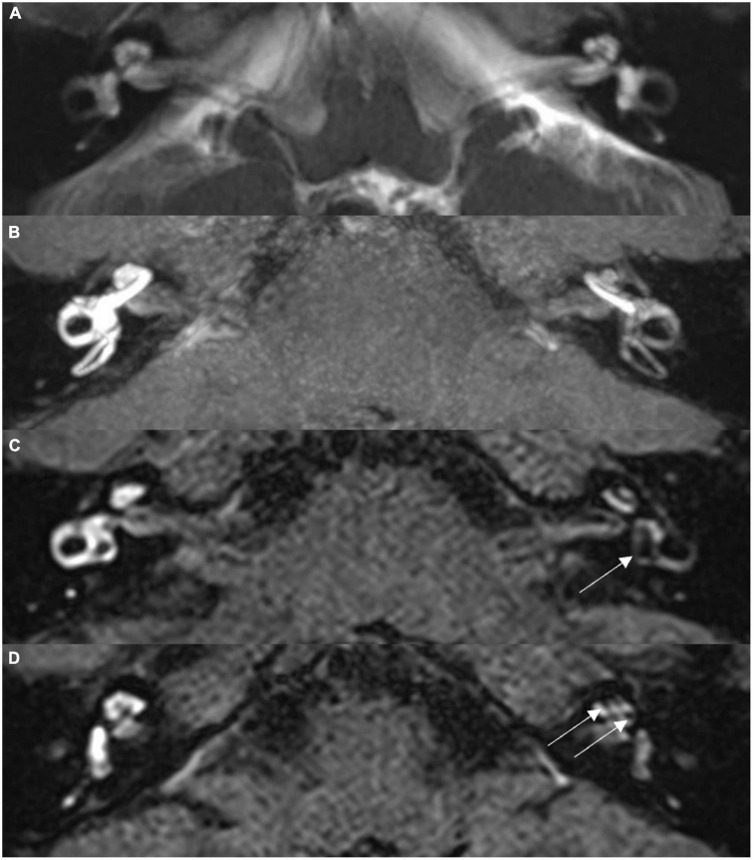
Magnetic resonance imaging (MRI) scans for a 36-year-old male with left-sided Ménière’s disease. **(A)** Routine MRI hydrography image, maximum intensity projection, showing symmetrical signal for bilateral labyrinth structures, without abnormality. **(B)** Isotropic 3D-SPC inversion recovery FLAIR image, maximum intensity projection reconstruction, showing narrowed left cochlear canal and significantly low signal in the vestibule. **(C)** Thin section original image from scan **(B)**; arrow indicates dilated sacculus and utriculus. **(D)** Thin section original image from scan **(B)**; arrow indicates the expanded scala media of the cochlea.

Exemplary MR images are shown in Figure 1. Figure 1A presents routine hydrography image. 3D-FLAIR images are shown in Figures 1B–D, in which the high signal area represented Gd in the perilymphatic space, whereas the low signal area represented the endolymphatic space without Gd. For the MD affected side, cochlear ELH was rated as none in 16 cases (22.86%), mild in 51 cases (72.86%), and significant in 3 cases (4.29%). No vestibular ELH was identified in 43 cases (61.43%), mild in 24 cases (34.29%), and significant in 3 cases (4.29%). As for the contralateral side, cochlear ELH was rated as none in 63 cases (90%) and mild in 7 cases (10%). No vestibular ELH was found in 63 cases (90%) and mild in 7 cases (10%).

A total of 51 patients underwent ECochG. Among the affected ears, positive results were yielded in 29 (56.86%), negative in 15 (29.41%), and no clear waveforms could be elicited in 7 (13.73%). Forty-four patients completed the glycerol test. Twenty-two patients (50%) exhibited positive results and 22 (50%) negatives. A total of 65 patients underwent caloric test. Caloric response was normal in 24 (36.92%) cases. Thirty-six patients (55.38%) had abnormal CP on the affected side, and 5 (7.69%) had bilateral vestibulopathy. cVEMP and oVEMP was performed in 51 and 28 cases, respectively. For the MD affected ears, abnormal response was identified in 24 (47.06%) for cVEMP and 10 (35.71%) for oVEMP, respectively. Twenty-nine patients underwent vHIT, of which 3 cases (10.34%) showed pathological results.

### 3.2. Agreement between radiological ELH and neurotological findings in MD patients

The agreement between audio-vestibular results and radiological ELH in the cochlear or vestibular compartment was shown in Table 2. Those patients who failed to elicit identifiable waveforms in ECochG were excluded from statistical analysis. The positive agreement rate (55.4%) and overall agreement rate (66.2%) between caloric test and radiological ELH were the highest. Overall poor negative agreement was noted between radiological ELH and neurotological tests, with vHIT being the highest (24.1%). Kappa test showed slight agreement between Gd-enhanced MRI and caloric test, cVEMP, oVEMP and vHIT (kappa value = 0.189, 0.093, 0.140, 0.071, respectively). Poor agreement existed between Gd-enhanced MRI and ECochG (kappa value = −0.081), and glycerol test (kappa value = −0.045). The incidence of ELH revealed by Gd-enhanced MRI was significantly higher than that of pathological findings in glycerol test (39/44, 88.6%; 22/44, 50%; *p* < 0.001), caloric test (53/65, 81.5%; 41/65, 63.1%; *p* = 0.017), cVEMP (42/51, 82.4%; 24/51,47.1%; *p* < 0.001), oVEMP (22/28, 78.6%; 10/28, 35.7%; *p* = 0.002), and vHIT (22/29, 75.9%; 3/29, 10.3%; *p* < 0.001). The incidence of radiological ELH and pathological ECochG was not significantly different (33/44, 75%; 29/44, 65.9%; *p* = 0.503).

**TABLE 2:** Agreement of audio-vestibular results and radiological ELH of cochlear and/or vestibular in patients with unilateral MD.

	Positive agreement rate (%)	Negative agreement rate (%)	Overall agreement rate (%)	*P*-value	Kappa value
ECochG	47.7	6.8	54.5	0.503	−0.081
Glycerol test	43.2	4.5	47.7	<0.001	−0.045
Caloric test	55.4	10.8	66.2	0.017	0.189
cVEMP	41.2	11.8	53.0	<0.001	0.093
oVEMP	32.1	17.9	50.0	0.002	0.140
vHIT	10.3	24.1	34.4	<0.001	0.071

ELH, endolymphatic hydrops; MD, Ménière’s disease; ECochG, electrocochleogram; cVEMP, cervical vestibular evoked myogenic potential; oVEMP, ocular vestibular evoked myogenic potential; vHIT, video head impulse test.

### 3.3. Correlation between the degree of radiological ELH and clinical features in MD patients

The correlation between the patient’s imaging ELH in cochlear and vestibular compartment and clinical characteristics and audio-vestibular tests results was shown in Table 3, respectively. The degree of cochlear hydrops was positively correlated with the pure tone average (PTA) in the affected side (*r* = 0.2679, *p* = 0.0249). The degree of vestibular hydrops was positively correlated with the duration of disease (*r* = 0.2592, *p* = 0.0303), the PTA in the affected side (*r* = 0.2728, *p* = 0.0223) and the glycerol test results (*r* = 0.3944, *p* = 0.0061).

**TABLE 3:** Correlation between the clinical feature and degree of radiological ELH in cochlear or vestibular in patients with unilateral MD.

	Degree in cochlear hydrops		Degree in vestibular hydrops	
	**Correlation coefficient (r)**	***P*-value**	**Correlation coefficient (r)**	***P*-value**
Age	−0.0415	0.7328	−0.1846	0.1261
Duration	0.1635	0.1761	0.2591	0.0303
Ménière’s stage	0.1157	0.3401	0.2102	0.0807
PTA	0.2679	0.0249	0.2728	0.0223
SP/AP	−0.1174	0.4477	0	1
Glycerol results	0.0149	0.9208	0.3944	0.0061
CP-value	0.0405	0.7588	0.0734	0.5774
cVEMP	−0.0821	0.5667	−0.0397	0.7822
oVEMP	0.2099	0.2837	0.1076	0.5858
vHIT	−0.0222	0.9141	−0.0752	0.715

ELH, endolymphatic hydrops; MD, Ménière’s disease; PTA, pure tone average; SP, summating potential; AP, action potential; CP, canal paresis; cVEMP, cervical vestibular evoked myogenic potential; oVEMP, ocular vestibular evoked myogenic potential; vHIT, video head impulse test.

## 4. Discussion

### 4.1. Enhanced MRI of the inner ear is superior to the auditory-vestibular tests to identify ELH

Our results showed that, in the affected ears of patients with unilateral MD, the incidence of ELH demonstrated by inner ear MRI was higher than that of pathological findings (or positive results) in audio-vestibular tests except for the pure tone audiometry, indicating that MRI of the inner ear with Gd enhancement was more sensitive in detecting ELH compared with neurotological evaluations.

Nowadays, the diagnosis of MD is primarily clinical, which mainly relies on typical manifestations of recurrent vertigo attacks, fluctuating hearing loss, tinnitus, and aural fullness and the pure-tone audiometric findings. Radiological and audio-vestibular evaluations other than pure-tone audiogram have not yet been included in most diagnostic criteria (Lopez-Escamez et al., 2015; Basura et al., 2020). Traditional audiometric test for identifying ELH, including click evoked ECochG and audiometric glycerol test, have a relatively low sensitivity of about 60%. Moreover, the application of these tests may be restricted in certain situations, for example, no discernable ECochG waveforms could be obtained in case of severe-to-profound hearing loss, and occasionally some patients are intolerant or allergic to glycerol ingestion. Fukuoka et al. (2012) evaluated the diagnostic performance of Gd-enhanced MRI of the inner ear for identifying MD and reported highest sensitivity (95.0%) by Gd-enhanced MRI, followed by transtympanic click ECochG (60.0%) and glycerol testing (55.0%). In a systemic review, Ziylan et al. (2016) demonstrated that Gd-enhanced MRI of the inner ear yields higher sensitivity than traditional click ECochG for detecting ELH in patients with definite MD. Recently, the sensitivity of ECochG was enhanced by using transtympanic electrode and tone burst stimuli (Gibson, 2009), leading to better performance in diagnosing ELH (Hornibrook et al., 2015; Hornibrook, 2017). Altogether, these results emphasize high diagnostic value of the Gd-enhanced MRI of the inner ear in patients with MD. Although not yet essential for diagnosis, radiological evidence of ELH is still important for understanding the pathogenic mechanisms of MD and may potentially be incorporated into the future diagnostic criteria.

### 4.2. Relationship between audio-vestibular test results and radiological ELH grade of inner ear

In our study, we found that radiological evidence of ELH *in vivo* did not parallel with neurotological functional findings. This may be due to that other than direct morphological evidence of ELH, the neurotological assessment probes the functional status of the inner ear end organs as well as the integrity of their associated neural pathways. And the results may be affected by a variety of confounding factors, such as stimulus frequency, recording method, age, fluctuating nature of MD, etc. For instance, SP/AP ratio measured by ECochG has been deemed as indication of cochlear ELH, which is assumed to be caused mainly by the displacement of the basilar membrane toward the scala tympani. However, this enhanced SP in MD might also reflect the malfunction of the hair cells rather than ELH (Takeda and Kakigi, 2010). Cervical and ocular VEMP could evaluate the otolith-colic and otolith-ocular reflex, respectively. MD patients typically show lower response prevalence, smaller amplitudes, higher thresholds as well as altered frequency tuning (Murofushi, 2016). Abnormal neurotransmitter modulation originating in the brainstem may also interfere with otolithic function and its associated neural pathways (Allena et al., 2007; Hamel, 2007). As for evaluating function of semicircular canal (SCC), the instrumental tests mainly include the caloric test and vHIT. It has been reported that MD patients tend to have a dissociated pattern of caloric-vHIT response (McCaslin et al., 2015; Rubin et al., 2018; Zhou et al., 2020; Hannigan et al., 2021). The exact mechanism underlying this caloric-vHIT dissociation remains to be determined. McGarvie et al. (2015) proposed that the dilated semicircular duct in hydropic labyrinths could result in local convective flow, which dissipates thermally induced hydrostatic pressure across cupula, while exert little effect on responses to rotation. An alternative explanation is the selective damage of type II vestibular hair cell population in MD and differential frequency stimulation of caloric test and vHIT (Tsuji et al., 2000). Furthermore, MD patients may exhibit a variety of abnormal vHIT response, including enhanced gain (Curthoys et al., 2021), saccades with normal gain (Jerin et al., 2019), or fluctuating response (Yacovino et al., 2017), etc.

#### 4.2.1. Hearing level and the ELH grade in the cochlear and vestibule

In our study, both cochlear and vestibular hydrops was positively proportional to PTA in the affected side. Previous literature consistently demonstrated positive correlations between cochlear ELH grades and pure tone hearing level, especially in the low frequency range (Gürkov et al., 2011; Cho et al., 2018; Yang et al., 2018; Sluydts et al., 2021). The number of hydropic cochlear sites were significantly correlated with MD stage graded by PTA threshold (Fiorino et al., 2011). These findings were compatible with the histopathological observations that the severity of ELH was associated with the level of hearing impairment and duration of the disease (Okuno and Sando, 1987). However, this association remains controversial (Xie et al., 2021). Gürkov et al. (2016) argued that the severity of ELH was not always parallel with the clinical symptoms, as hearing can be preserved despite marked ELH.

As for the relationship between vestibular ELH and PTA, although it seems counterintuitive, similar findings have also been shown in several other studies. Using a quantitative approach, Sepahdari et al. (2015) showed that vestibular ELH correlated with the severity of hearing loss. Similarly, vestibular ELH significantly correlated with PTA threshold (Yang et al., 2018). Sluydts et al. (2021) demonstrated positive correlations between vestibular ELH grades and pure tone hearing level, especially in the low frequency range. In contrast, Xie et al. (2021) found no association between vestibular ELH and hearing levels.

As vestibular ELH was strongly correlated with the cochlear ELH (Cho et al., 2018), presumably, vestibular ELH grade might be associated with PTA in the affected side through its association with cochlear ELH and is not an independent variable. The association between vestibular ELH and PTA might also be explained by the involvement of saccule. Compared to utricular ELH, saccular ELH was more prevalent and more prominent (Cho et al., 2021), thus making itself a major contributor to vestibular ELH. Recent radiological studies using SURI (the inverted ratio between the utricle and saccule) criteria seem to support this hypothesis. Attyé et al. (2018) suggested that saccular hydrops mainly reflects the level of hearing impairment, even regardless of MD. Quatre et al. (2019) also showed that patients with MRI-demonstratable vestibular ELH suffered from greater hearing loss compared with those without. It has been speculated that saccule plays a buffering role in endolymph resorption. When the endolymphatic pressure exceeds the limit of saccular compliance, the endolymph regulation is disturbed, resulting in cochlear hydrops, and subsequent cochlear damage occurs (Salt and Plontke, 2010).

#### 4.2.2. Disease duration and the ELH grade in the vestibule

In this study, we identified a significant association between MD duration and degree of vestibular ELH, which suggested that MD patients were experiencing deterioration of ELH as the disease progressed. This gradual expansion of endolymphatic space suggested that ELH is probably resulted from progressive degeneration related to advanced disease. Until now, the association between MD duration and ELH grade in the vestibule remains controversial. Fiorino et al. (2011) exhibited that the number of involved vestibular sites were significantly associated with MD duration. And a significant correlation between MD duration and ELH both in cochlear and vestibule has been also demonstrated (Wu et al., 2016). Nevertheless, some studies found no association between vestibular hydrops and disease duration (Yang et al., 2018; Xie et al., 2021; Jasińska et al., 2022). Xie et al. (2021) attributed this lack of correlation to the heterogeneity of the disease severity with disease progression, as the speed of deterioration can vary substantially across individuals.

#### 4.2.3. Caloric reflex and the ELH grade in the vestibule

The relationship between the caloric reflex and the extent of vestibular ELH are variable. Our results revealed no correlation between the extent of vestibular ELH and caloric response, which is consistent with several other studies. Jerin et al. (2018) found no significant relationship between cochlear or vestibular ELH and CP. Kato et al. (2011) reported no significant correlation between the degree of ELH in the horizontal SCC and the presence of CP, the CP value or the SPV_max_ of caloric response. Vestibular hydrops tended to deteriorate as the caloric paresis increased, but this correlation was statistically insignificant (Gürkov et al., 2011). However, opposite results have been yielded in other studies. By using quantitative measure of hydrops, Choi et al. (2017) and Cho et al. (2018) demonstrated that in the affected ear of MD, the ratio of vestibular hydrops correlated with mean SPV_max_ of caloric nystagmus following warm and cold irrigation and the CP value was significantly associated with relative ratio of vestibular hydrops.

According to the hydrostatic temperature dissipation hypothesis, dilated endolymphatic space in horizontal SCC could increase endolymphatic turbulence within the duct, therefore, the thermally induced pressure gradient across the cupula and cupula displacement was diminished (McGarvie et al., 2015). Therefore, vestibular ELH may theoretically impact the caloric response in the affected ear. However, this hypothesis was challenged by the histopathological observation that no significant hydropic dilation of SCC was present on the affected sides in MD patients (Cho et al., 2021). Alternatively, herniation of the enlarged saccule and/or utricle into the horizontal SCC was quite common (Cho et al., 2021), and has been reported to correlate with impaired caloric responses and progressive ELH (Gürkov et al., 2012; Cho et al., 2018; Sugimoto et al., 2018). It is speculated that the impaired caloric response in MD patients resulted from vestibule herniation, rather than expansion of the endolymphatic space within the canal. This inconsistent relationship between caloric response and vestibular ELH may arise from the following reasons. Firstly, compared to significant ELH in the saccule or utricle, ELH in the horizontal SCC was less common and with variable degree (Schuknecht and Gulya, 1983). Secondly, extremely severe vestibular ELH may impede the passage of contrast agent into the SCC due to the blockade of perilymphatic communication from the oval and round window to the SCC, or obscure the observation of ELH in the horizontal SCC due to the anatomical proximity of utricle and the ampulla of horizontal SCC (Nakashima et al., 2009a). Thirdly, the current ELH grading systems, such as Nakashima criteria (Nakashima et al., 2009b), Bernaerts criteria (Bernaerts et al., 2019), or Baráth criteria (Baráth et al., 2014), did not allow for direct evaluation of ELH in SCCs.

#### 4.2.4. Audiometric glycerol test and the ELH grade in the vestibule

In this study, MD patients with severe vestibular ELH tended to have positive glycerol test results. Dehydrating agents combined with audio-vestibular test, including pure tone audiometry, otoacoustic emissions, VEMP, etc, are considered to reveal ELH in different compartments of the inner ear. To date, few studies have addressed the association between dehydration test and the MRI-demonstrable ELH in patients with MD. Shiraishi et al. (2020) found that the prevalence of saccular ELH was 55% by using furosemide-loading cVEMP, and this finding was more closely related to the results of MRI-demonstrable ELH in the cochleae. Recently, Wang et al. (2020) observed higher baseline level of cochlear ELH in MD patients with positive audiometric glycerol test results, indicating that the results of audiometric glycerol test correlate with the level of ELH in the cochlea, which is discrepant with our findings. Future investigations are needed to elaborate the relationship between compartmental ELH and dehydrating agents combined with specific functional inner ear test, such as VEMPs, ECochG, otoacoustic emissions, etc.

There were several limitations in this study. Firstly, due to the retrospective nature of this study, not all participants completed a comprehensive battery of auditory-vestibular functional tests, except for pure tone audiometry, thus might bias the results. Secondly, our study used intratympanic injection as the routine route for Gd administration, because of the technical simplicity and with an attempt to avoid potential impairment of renal function. Diffusion of Gd by means of intratympanic injection into the perilymph may be insufficient, possibly due to fibrosis or obstruction of round and oval window (Yoshioka et al., 2009), Gd leakage from the tympanic puncture or the eustachian tube, or uneven distribution to the distal parts of the membranous labyrinth (e.g., SCCs) (Sun et al., 2021), thus yielding false-positive results. Conversely, intravenous Gd administration yields a weaker MRI signal but a more uniform distribution and allows for simultaneous evaluation of bilateral inner ear. Thirdly, we included the classic Nakashima grading scale in our routine diagnostic workup, as it is most commonly used in the current literature. However, this grading system visually evaluates ELH based on only two representative imaging sections of the cochlea and vestibule, and the boundary between endolymphatic and perilymphatic compartment is delineated by the operator, which makes the result less accurate and less objective. Recently, several new grading systems have been proposed, such as the semi-quantitative SURI grading system (Attyé et al., 2017) and the 3D MRI-based quantitative volumetric measurement of the endolymphatic space (Inui et al., 2021). Therefore, further prospective studies involving larger sample-size with correlation to clinical, audio-vestibular, and radiological investigations are called for to comprehensively evaluate the severity of disease and to optimize the MRI grading system in MD patients.

## 5. Conclusion

In the diagnosis of MD, contrast-enhanced MRI of the inner ear is advantageous in detecting ELH over the conventional audio-vestibular evaluations, which estimates more than hydropic dilation of endolymphatic space.

## Data availability statement

The original contributions presented in this study are included in the article/Supplementary material, further inquiries can be directed to the corresponding authors.

## Ethics statement

The studies involving human participants were reviewed and approved by the Ethical Committee of Union Hospital, Tongji Medical College of Huazhong University of Science and Technology. Written informed consent to participate in this study was provided by the participants’ legal guardian/next of kin.

## Author contributions

WF, RZ, JL, and HW performed the material preparation and data collection. YaL, YiL, KX, and HM performed the data analysis and interpretation. YaL and BL wrote the first draft of the manuscript. HM and BL performed the critical review of the manuscript. All authors contributed to the study conception and design, read, and approved the final manuscript.
