# Supplementary material for: Comparison between audio-vestibular findings and contrast-enhanced MRI of inner ear in patients with unilateral Ménière’s disease
Source: Front Neurosci. 2023 Mar 13;17:1128942. doi: 10.3389/fnins.2023.1128942 (PMC10040662; doi:10.3389/fnins.2023.1128942)
Supplement: Supplementary file 1 [file Table_1.DOCX]

| **Supplementary Table 1**: MR scanning protocol | | | |
| --- | --- | --- | --- |
| Scanning Parameters（3.0T） | T2-TSE | 3D-t2-SPACE | 3D-SPACE-FLAIR |
| Plane | coronal | axial | axial |
| TR (ms) | 6000 | 6000 | 6000 |
| TE (ms) | 96 | 132 | 388 |
| TI (ms) | / | / | 2100 |
| Slice thickness(mm) | 3 | 0.5 | 0.5 |
| Slice number | 48 | - | - |
| Scan time (seconds) | 72 | 288 | 384 |
| Voxel size (mm^3^) | 3 x 1 x 1 | 0.5 x 0.5 x 0.5 | 0.5 x 0.5 x 0.5 |
| 3D-SPACE: Three-dimensional sampling perfection with application optimized contrasts using different flip angle evolutions; 3D-FLAIR: three-dimensional fluid-attenuated inversion recovery; TR: Repetition Time; TE: Echo Time; TI: Inversion time. | | | |
|  |  |  |  |
|  |  |  |  |
